# Novel Record Replacement Algorithm and Architecture for QoS Management over Local Area Networks

**DOI:** 10.3390/mi13040594

**Published:** 2022-04-10

**Authors:** Yi-Chih Tung, Yuk-Wing Law, Wen-Jyi Hwang, Tsung-Ming Tai, Chih-Hsiang Ho, Cheng-Chang Chen

**Affiliations:** 1 Department of Electronic Engineering, Ming Chi University of Technology, New Taipei City 243, Taiwan; iggy@mail.mcut.edu.tw; 2 Department of Computer Science and Information Engineering, National Taiwan Normal University, Taipei 116, Taiwan; 60847037s@ntnu.edu.tw; 3 NVIDIA AI Technology Center, Taipei 114, Taiwan; ntai@nvidia.com; 4 Institute for Information Industry, Taipei 106, Taiwan; andrew.ho@iii.org.tw; 5 Bureau of Standards, Metrology and Inspection, M.O.E.A., Taipei 100, Taiwan; chang.chen@bsmi.gov.tw

**Keywords:** System-on-Chip, Quality-of-Service, field programmable gate array, local area network, general regression neural network, network function virtualization

## Abstract

An effective System-on-Chip (SoC) for smart Quality-of-Service (QoS) management over a virtual local area network (LAN) is presented in this study. The SoC is implemented by field programmable gate array (FPGA) for accelerating the delivery quality prediction for a service. The quality prediction is carried out by the general regression neural network (GRNN) algorithm based on a time-varying profile consisting of the past delivery records of the service. A novel record replacement algorithm is presented to update the profile, so that the bandwidth usage of the service can be effectively tracked by GRNN. Experimental results show that the SoC provides self-aware QoS management with low computation costs for applications over virtual LAN.

## 1. Introduction

Basic Internet services are usually delivered on a best effort basis, without taking any quality requirements into consideration. To satisfy the demands of applications and users in the network, Quality-of-Service (QoS) management [1,2] is usually employed by allocating existing resources to Internet services. A challenging issue for QoS management is the efficient utilization of network resources by the integration of a variety of hardware and software appliances. Network resources may not be effectively exploited by traditional QoS approaches such as the ones designed for peak requirements. Therefore, they are inefficient to cope with current diversified communication traffic demands.

Software-defined networking (SDN) [3] is a technique that provides programmability in configuring network resources. The SDN technique offers a valuable mechanism for dynamic and cost-effective network management. In addition, the SDN can be incorporated into the network function virtualization (NFV) [3,4], by which virtual network functions (VNFs) are interconnected into different delivery operations. For applications based on the 5G network and beyond [5] such as eHealth, smart poles, and smart cities [6,7,8], SDN and NFV for QoS could play important roles for efficient allocations of network resources for communication services.

The study in [9] builds a virtual local area network (LAN) integrating SDN with NFV, where both the service quality prediction and subscription schemes are implemented as VNFs in the virtual LAN. The major goal of quality prediction schemes is to forecast the network resources required for satisfying the prescribed QoS level for a network service via the general regression neural network (GRNN) [10] algorithm. The prediction could be based on the profile containing delivery records of the past bandwidth usage of the service and the corresponding feedback. The subscription of the service is then carried out from the prediction results. A drawback of the system is the high computational complexities for service quality prediction. This may introduce long latency for updating the subscription of the service.

An approach for solving the latency issues for QoS management is the employment of a hardware accelerator for NFV. A field programmable gate array (FPGA) [11,12] implementation for smart QoS management is proposed in [13]. The hardware architecture is able to accelerate the self-aware quality prediction based on GRNN. However, the updating operations for QoS management are achieved only by appending more delivery records to the profile for GRNN prediction. As the profile size reaches the upper bound affordable by the hardware, record replacement is necessary. There is no hardware-based replacement strategy for the system. The systems based on simple random selection strategy for replacement may not be self-aware for maintaining high prediction accuracy.

The objective of this paper is to present a novel system-on-chip (SoC) with record replacement for self-aware QoS management over a virtual LAN. An FPGA architecture of the GRNN algorithm is proposed as a hardware VNF for the prediction of delivery quality for a service. Based on the prediction results, optimal bandwidth allocation is performed to the service so that the service can be delivered with desired quality. The VNFs for traffic control operating in conjunction with the proposed FPGA-based QoS management VNFs are also implemented for the virtual LAN.

In addition to providing fast computation for producing prediction results for delivery quality, the proposed FPGA architecture contains a dedicated circuit for profile updating. Apart from basic record appending and removal operations, the dedicated circuit support online record replacement based on a novel record replacement algorithm. In this way, the profile for GRNN prediction can be effectively updated even for a small profile buffer. In the algorithm, the past records are separated into two groups: positive response group and negative response group. A record with positive response implies the delivery is achieved with satisfactory quality. Conversely, a negative response record reveals that the quality of the delivery is below expected level. One of the groups will be dynamically identified as the insignificant group based on the most recently received record. In the insignificant group, the oldest record is removed.

Both analytical and numerical evaluations are provided for the algorithms and systems presented in this study. Analytical results show that the GRNN-based prediction with the proposed record replacement algorithm achieves self-aware prediction. Furthermore, numerical evaluations reveal that the proposed smart SoC system offers accurate quality prediction for services for the efficient bandwidth allocation of the virtual LAN. All the results reveal that the proposed smart FPGA-based SoC is effective for the exploitation of network resources for dynamic and self-aware QoS management.

The remaining parts of this paper are organized as follows. Section 2 provides a brief overview of the works related to this study. The proposed QoS management algorithm and its profile updating techniques are presented in Section 3 and Section 4, respectively. The SoC implementation supporting the QoS management with profile updating is then presented in Section 5. Section 6 shows some experimental results and evaluations. Concluding remarks are given in Section 7.

## 2. Related Works

A number of neural networks, such as multilayer perceptron (MLP) and recurrent neural network (RNN) [14,15,16], can be effectively used for the prediction of delivery quality for a service. However, offline training is required prior to the deployment of networks. For a new service, without a long collection of the corresponding delivery records, it would be difficult to find sufficient training data for accurate quality prediction. Therefore, a long delay would be necessary for a new service before an effective QoS management can be carried out.

The auto-regressive integrated moving average (ARIMA) [17] and GRNN [9] can be employed for delivery quality prediction without offline training. Similar to the approaches based on MLP and RNN, the ARIMA performs the prediction based only on the past source data rates. Because the bandwidth usage of a service may not be stationary, it would be difficult to maintain high prediction accuracy in the presence of surges or plummets in the source data rate of the service. To solve the nonstationary issues, a time-varying profile is used for GRNN-based prediction [9]. In addition to bandwidth allocations, the profile also contains the corresponding service responses. Profile updating policies are proposed for accommodating new service responses, so that the algorithm can be self-adaptive to new trends for the service.

A drawback for the GRNN-based prediction is the high computational complexities because of the employment of Gaussian kernels. One approach to accelerate the computation is the employment of FPGA techniques. Because of high flexibility and high computation speed, FPGA has been found to be effective for the hardware VNF implementations [18]. Examples of the FPGA implementations include the deep packet inspection and firewall [19]. A number of FPGA architectures [20,21,22] have been proposed for accelerating GRNN computation. However, many FPGA architectures are targeted for pattern classification applications with a fixed profile. Direct employment of the architectures for QoS management would then be difficult.

The GRNN prediction in [13] is implemented as a hardware VNF for smart QoS management. However, the hardware VNF does not address the record replacement issue after the buffer for the record collection becomes full. Although simple approaches such as random replacement are possible, prediction performance may be degraded because of the possible removal of important records. The least significant record removal policy proposed in [9] could be adopted. However, the policy is based on full-search operations with high computational complexity. This could impose a heavy computational load for the QoS management system. To achieve online self-adaptive and self-aware QoS management, a dedicated circuit for fast record replacement in the profile is desired.

## 3. Proposed QoS Management Algorithm

This section covers the infrastructure for the QoS server, QoS level definition, GRNN-based quality prediction, and the proposed QoS management algorithm in detail. To facilitate an understanding of the proposed algorithms, Appendix A includes a list of frequently used symbols.

### 3.1. Infrastructure for QoS Server

**Figure 1:**
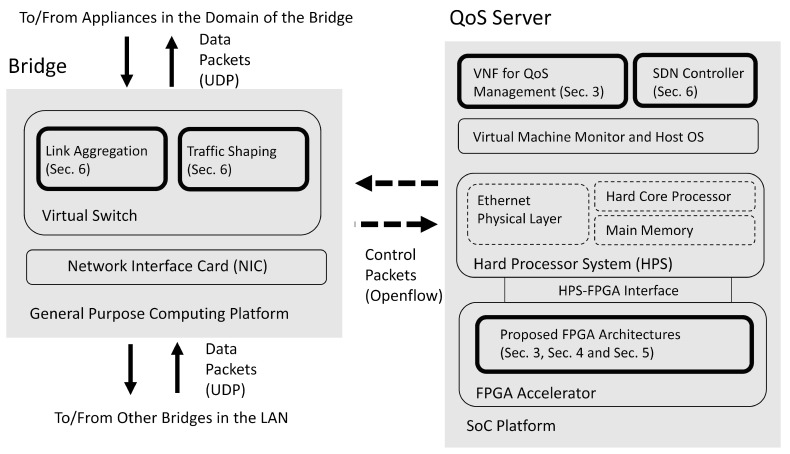
The block diagrams of a bridge and a QoS server in the virtual LAN. The bridge is a general purpose computer with a virtual switch. The QoS server is an SoC consisting of FPGA and HPS. In this study, the highlighted blocks are implemented.

For the virtual LAN considered in this study, there are two or more domains. A multi-link core network is responsible for the communication among different domains. Only the bridges in each domain are connected to the core network. There is a QoS server in the LAN for QoS management. The bridges carry out data forwarding operations subject to the constraint of the bandwidth allocated by a QoS server. The block diagrams of a bridge and a QoS server in the virtual LAN are shown in Figure 1.

We can see from Figure 1 that dedicated FPGA circuits are implemented as accelerators for the VNFs in the SoC for QoS management. In this way, the latency for QoS management can be effectively reduced. The FPGA-assisted SoC can be separated into two portions: hard processor system (HPS) and FPGA accelerator. The HPS contains a hard core processor, a main memory, and an Ethernet physical layer. The HPS is responsible for delivering control packets between the QoS server and a bridge. The delivery of control packets is based on the Openflow protocol. The HPS operates with the FPGA accelerator through HPS-FPGA interface. The FPGA accelerator carries out the GRNN-based quality prediction and the proposed profile updating algorithms in the SoC.

The bridges in the LAN can operate in a general-purpose computing platform. It contains a virtual switch supporting link aggregation and traffic shaping based on the commands from QoS server by the Openflow protocol. In addition, each bridge supports the delivery of data packets to/from the other bridges in the LAN by user datagram protocol (UDP). In Figure 1, the components developed by this study are highlighted. We have also marked the corresponding sections for the highlighted components.

### 3.2. QoS Level

In this study, we define a service as a dataflow between two appliances from different domains. The service is delivered subject to a QoS level, which is dependent on the redundant bandwidth reserved for a service. Let $x=\{x_{1},\;\ldots ,\;x_{n}\}$ be the bandwidth allocation to the service, where $x_{j}$, j=1,…,n, is the bandwidth of link *j* reserved for the service, and *n* is the number of links in the core network. Let

(1) $$\left|x\right|=\sum_{j=1}^{n}x_{j}$$

be the bandwidth allocated by the QoS server. Let *R* be the actual source data rate of the service. Note that *R* and |x| may not be identical. When |x|≥R, we define

(2) RAB=|x|−R

as the residual allocation bandwidth (RAB) for data delivery, which can be regarded as the unused network resources for the service. Conversely, when |x|<R, let

(3) DLR=R−|x|

be the data loss rate (DLR) of the service because of the lack of bandwidth. The RAB and DLR are the basic performance metrics for QoS management. Based on RAB and DLR, we define the Extended RAB (ERAB) as

(4) $$\mathrm{ERAB}=\left\{\begin{array}{cc} \mathrm{RAB} & \mathrm{when}\;|x|\geq R,\\ -\mathrm{DLR} & \mathrm{when}\;|x|<R. \end{array}\right.$$

Clearly, when ERAB in (4) is positive, the service is not able to utilize all the available bandwidth. In contrast, the service needs more network bandwidth when a negative ERAB is observed. The ERAB could be regarded as useful feedback information for the service. In this study, an approach based on quantized ERAB is adopted for QoS management. Let *L* be the number of quantization levels. Based on *L*, let $I_{k}\subset R$, k=0,…,L−1, be a set of ERAB intervals defined as

(5) $$I_{k}=\left\{\begin{array}{cc} \left(-\infty ,\;\eta_{1}\right] & \mathrm{when}\;k=0,\\ \left(\eta_{k},\;\eta_{k+1}\right] & \mathrm{when}\;k=1,\;\ldots ,\;L-2,\\ \left(\eta_{L-1},\;\infty \right] & \mathrm{when}\;k=L-1, \end{array}\right.$$

where $\left\{\eta_{1},\;\ldots ,\;\eta_{L-1}\right\}$ is a set of thresholds satisfying $\eta_{i}<\eta_{j}$ for i<j. The output of the quantizer, denoted by y, is given by

(6) $$\begin{array}{cc} y=k & \mathrm{when}\;\mathrm{ERAB}\in I_{k}. \end{array}$$

In the proposed algorithm, the quantization result y is regarded as a service quality. Table 1 shows an example of six service qualities (i.e., L=6) and the corresponding ERAB intervals. From (4), the ERAB can be regarded as the redundant bandwidth reserved for a service. Therefore, a positive service quality (i.e., y>0) has redundant bandwidth. A positive service quality with a large y value would provide a large reserved network resource for a service for the accommodation of unexpected increases in the source data rate. It is then beneficial for maintaining low DLR for the service. On the contrary, there may be no redundant bandwidth for the service quality with y=0. Furthermore, the bandwidth shortage of a negative service quality is likely, so that packet losses are possible.

In the proposed algorithm, it is necessary to specify a QoS level before QoS management. The QoS level can be determined from the requirements for the service. One simple approach to designate a QoS level is to set the constraint on the lower bound *T* of expected service qualities for the data delivery, where 0<T≤L−1. Therefore, QoS levels with higher *T* values imply better service qualities. Given the quantizer in (6), there are (L−1) QoS levels for QoS management. As a result, the number of QoS levels supported by the proposed QoS management scheme would grow with *L*. It provides larger flexibilities as compared with the studies in [9], where only a fixed number of QoS levels are considered.

Table 2 shows an example of a set of QoS levels based on the service qualities defined in Table 1. It can be observed from Table 2 that QoS levels with higher *T* values allow fewer ERAB intervals for the service. In particular, for the delivery of a service with the highest QoS level (i.e., T=(L−1)=5), the goal of the delivery is only to maintain ERAB values in the interval $I_{5}=\left[11.25,\;\infty \right)$.

### 3.3. GRNN-Based Service Quality Prediction

Let B be the set of bandwidth allocations provided by the core network of the LAN for the service. It is given by

(7) $$B=\{x:x_{j}=k_{j}\Delta ,\;0\leq x_{j}\leq B_{j},\;1\leq j\leq n\},$$

where $B_{j}$ is the maximum allowed bandwidth at the link *j* for the service, Δ>0 is the step size, $k_{j}\geq 0$ is an integer. For each bandwidth allocation x∈B, we carry out the service quality prediction.

Let $P=\{\left(x_{i},y_{i}\right),\;i=1,\;\ldots ,\;p\}$ be a profile containing *p* records of past services, where $\left(x_{i},y_{i}\right)$ is the *i*-th record consisting of bandwidth allocation $x_{i}$ and the corresponding service quality $y_{i}$. Based on the profile P, the GRNN is adopted for the service quality prediction. Given x and P, let $y^{\prime }$ be the result of the GRNN [10] computation. That is,

(8) $$y^{\prime }=\frac{\sum_{i=1}^{p}y_{i}W\left(x,x_{i}\right)}{\sum_{i=1}^{p}W\left(x,x_{i}\right)},$$

where

(9) $$\begin{array}{ccc} W(x,x_{i}) & = & \exp(\frac{-D(x,x_{i})}{\sigma^{2}}), \end{array}$$

(10) $$\begin{array}{ccc} D(x,x_{i}) & = & \sum_{j=1}^{n}{\left(x_{j}-x_{i,j}\right)}^{2}, \end{array}$$

and $x_{i,j}$ is the *j*-th element of $x_{i}$. Let $\hat{y}$ be the predicted service quality, which can be obtained from $y^{\prime }$ by a rounding operation as

(11) $$\hat{y}=\left\{\begin{array}{cc} L-1 & \mathrm{when}\;y^{\prime }\geq L-1.5,\\ k & \mathrm{when}\;k-0.5\leq y^{\prime }<k+0.5,\;\;k=1,\;\ldots ,\;L-2,\\ 0 & \mathrm{when}\;y^{\prime }<0.5. \end{array}\right.$$

Only the bandwidth allocations with $\hat{y}$ larger or equal to *T* are considered as candidates for the service. Let

(12) $$O=\{x:\hat{y}\geq T\}.$$

Let $x^{*}$ be the optimal bandwidth allocation in O, satisfying

(13) $$x^{*}=\min_{x\in O}\left|x\right|.$$

In the proposed algorithm, the $x^{*}$ is then served as the bandwidth allocated to the service. From (13), it can be observed that the search space O is required before the identification of $x^{*}$. To find the search space O, a full-search scheme for the computation of service quality prediction $\hat{y}$ over all elements in B may be necessary.

Algorithm 1 summarizes the operations of the proposed algorithm. As shown in Algorithm 1, each service based on the  bandwidth allocation $x^{*}$ of the current time slot results in a new service quality y. The profile P will then be updated after the new record $\left(x^{*},y\right)$ is available. It is not necessary to carry out a training process for profile updating. Only record appending or replacement operations are necessary. After the profile is updated, the new bandwidth allocation $x^{*}$ is determined for the next time slot. Detailed discussions of the profile updating are presented in the next section.
**Algorithm 1** The GRNN-based QoS Management Algorithm**Require:** Search space B. **Require:** Upper bound of profile size *C*. **Require:** Number of service qualities *L*. **Require:** The set of threshold $\left\{\eta_{1},\;\ldots ,\;\eta_{L-1}\right\}$ for determining service qualities. **Require:** Initial profile $P=\{x_{i},\;y_{i},\;i=1,\;\ldots ,\;p\}$. **Require:** QoS level specified by *T*. 1: **loop** 2:     **if** service required in new time slot **then** 3:         Compute the optimal bandwidth allocation $x^{*}$ from P by (13). 4:         Current time slot ← new time slot. 5:         Bandwidth allocation of current time slot $\leftarrow x^{*}$. 6:         Measure the ERAB defined in (4). 7:         Compute y from ERAB by (6). 8:         (P,p)←Profile_Update($x^{*}$, y, P, *p*, *C*, *T*) 9:         Wait till the end of the current time slot. 10:     **end if** 11: **end loop**


## 4. The Proposed Profile Updating Algorithm

To facilitate the presentation of the profile updating algorithm, we first define positive responses, negative responses, and self-aware QoS management. Given a QoS level *T*, a response y is said to be positive when y≥T. Otherwise, y is said to be negative. A QoS management algorithm is said to be self-aware when two conditions are met for a given service with QoS level *T*. Firstly, after a negative response is acquired, the algorithm will increase the total bandwidth allocated to the service. In addition, the algorithm will maintain or reduce the total allocated bandwidth after a positive response is obtained. In the remaining parts of this section, we show that the proposed profile updating algorithm has the advantage of being self-aware.

### 4.1. QoS Self-Awareness for Proposed GRNN Algorithm after Appending a New Record

Given a service with QoS level *T*, we can rewrite (12) for the search space O by (11) as

(14) $$O=\{x:y^{\prime }\geq T-1/2\}.$$

Because $y^{\prime }$ is dependent on the profile size *p* from (8), the set O is also dependent on *p*. Let O(p) be the set O with profile size *p*. By substituting (8) to (14), it can be derived that

(15) $$O\left(p\right)=\left\{x:\frac{\sum_{i=1}^{p}y_{i}W\left(x,x_{i}\right)}{\sum_{i=1}^{p}W\left(x,x_{i}\right)}\geq T-\frac{1}{2}\right\}.$$

We then rewrite (15) as

(16) $$O\left(p\right)=\left\{x:S_{1}+S_{2}\geq S_{3}\right\},$$

where

(17) $$S_{1}=\sum_{u=T}^{L-1}\sum_{i\in J_{u}}\left(u-T\right)W\left(x,x_{i}\right),S_{2}=\frac{1}{2}\sum_{i=1}^{p}W\left(x,x_{i}\right),S_{3}=\sum_{u=0}^{T-1}\sum_{i\in J_{u}}\left(T-u\right)W\left(x,x_{i}\right),$$

and

(18) $$J_{u}=\left\{i:1\leq i\leq p,y_{i}=u\right\}.$$

From (9), we see that $W(x,x_{i})\geq 0$. Therefore, It follows from (17) that $S_{1}\geq 0$, $S_{2}\geq 0$ and $S_{3}\geq 0$.

We next consider the scenario where the new record $\left(x^{*},y\right)$ is appended as the (p+1)-th record of the profile. In this case, there are p+1 records in the new profile. Therefore, the resulting set O is given by

(19) $$O\left(p+1\right)=\left\{x:\frac{yW\left(x,x^{*}\right)+\sum_{i=1}^{p}y_{i}W\left(x,x_{i}\right)}{W\left(x,x^{*}\right)+\sum_{i=1}^{p}W\left(x,x_{i}\right)}\geq T-\frac{1}{2}\right\}.$$

Two cases are then studied separately: a new positive response (i.e., y≥T) and a new negative response (i.e., y<T).

#### 4.1.1. New Positive Response

In this case, y≥T. Based on the similar approaches for obtaining (16) from (15), it can be shown from (19) that

(20) $$O\left(p+1\right)=\{x:S_{1}+S_{2}+\left(y-T+\frac{1}{2}\right)W\left(x,x^{*}\right)\geq S_{3}\},$$

where $S_{1}$, $S_{2}$ and $S_{3}$ are given in (17). Because $S_{1}>0$, $S_{2}>0$, $S_{3}>0$, and  y≥T, it can be easily shown that all the terms in (20) are positive. By comparing (16) with (20), we see that

(21) O(p+1)⊇O(p),wheny≥T.

From (13) and (21), it follows that

(22) $$|x^{*}\left(p+1\right)|\leq |x^{*}\left(p\right)|,\;\mathrm{when}\;y\geq T,$$

where $x^{*}\left(p\right)$ is $x^{*}$ when the size of profile P is *p*. Consequently, from (22), it can be observed that the proposed algorithm reduces the allocated bandwidth after a new positive response is obtained.

#### 4.1.2. New Negative Response

For the case of y<T, we can derive from (19) that

(23) $$O\left(p+1\right)=\{x:S_{1}+S_{2}\geq \left(T-y-\frac{1}{2}\right)W\left(x,x^{*}\right)+S_{3}\}.$$

Note that $S_{1}>0$, $S_{2}>0$, $S_{3}>0$, y<T, and y and *T* are integers. As a result, all terms in (23) are positive. It can then be concluded from (16) and (23) that

(24) O(p+1)⊂O(p),wheny<T.

Therefore,

(25) $$|x^{*}\left(p+1\right)|>|x^{*}\left(p\right)|,\;\mathrm{when}\;y<T.$$

Both (22) and (25) conclude the self-awareness of the GRNN-based QoS management algorithm.

### 4.2. QoS Self-Awareness for Proposed GRNN Algorithm after Replacing an Old Record

The profile size *p* grows as new records are acquired during the service. Therefore, for a service with long transmission, a large profile may be produced. This would increase the computation overhead for QoS management. One way to solving the issue is to maintain the profile size *p* as it reaches a predefined upper limit *C*. That is, when p=C, an old record $\left\{x_{q},y_{q}\right\}$, q∈{1,…,C}, is replaced by the new record $\left\{x^{*},y\right\}$. In this subsection, we investigate the conditions under which the selection of the old record $\left\{x_{q},y_{q}\right\}$ for the replacement would still attain the QoS awareness.

Because the replacement only occurs after the profile size *p* reaches *C*, let $O_{old}\left(C\right)$ and $O_{new}\left(C\right)$ be the set O(C) before and after replacement, respectively. In this operation, the old record $\left\{x_{q},y_{q}\right\}$ is replaced by new record $\left\{x^{*},y\right\}$. That is,

(26) $$O_{old}\left(C\right)=\left\{x:\frac{\sum_{i=1}^{C}y_{i}W\left(x,x_{i}\right)}{\sum_{i=1}^{C}W\left(x,x_{i}\right)}\geq T-\frac{1}{2}\right\},$$

and

(27) $$O_{new}\left(C\right)=\left\{x:\frac{yW\left(x,x^{*}\right)-y_{q}W\left(x,x_{q}\right)+\sum_{i=1}^{C}y_{i}W\left(x,x_{i}\right)}{W\left(x,x^{*}\right)-W\left(x,x_{q}\right)+\sum_{i=1}^{C}W\left(x,x_{i}\right)}\geq T-\frac{1}{2}\right\}.$$

We can rewrite (26) and (27) as

(28) $$O_{old}\left(C\right)=\left\{x:S_{1}+S_{2}\geq S_{3}\right\},$$

(29) $$O_{new}\left(C\right)=\left\{x:S_{1}+S_{2}+S_{4}\geq S_{3}\right\},$$

where $S_{1}$, $S_{2}$ and $S_{3}$ are given by (17), and 

(30) $$S_{4}=\left(y-\left(T-\frac{1}{2}\right)\right)W\left(x,x^{*}\right)+\left(\left(T-\frac{1}{2}\right)-y_{q}\right)W\left(x,x_{q}\right).$$

Two cases are also considered separately: a new positive response (i.e., y≥T) and a new negative response (i.e., y<T).

#### 4.2.1. New Positive Response

Consider a set M satisfying

(31) $$M=\{\left(x_{q},y_{q}\right):\left(x_{q},y_{q}\right)\in P,\;S_{4}>0,\;\forall x\}.$$

When a new positive response is received (i.e., y≥T), it is desired that the selected old record $\left(x_{q},y_{q}\right)$ to be replaced belongs to M so that $S_{4}>0$. From (28) and (29), it can then be concluded that

(32) $$O_{new}\left(C\right)⊇O_{old}\left(C\right),\;\mathrm{when}\;y\geq T\;\mathrm{and}\;(x_{q},y_{q})\in M.$$

Let $x_{old}^{*}\left(C\right)$ and $x_{new}^{*}\left(C\right)$ be the optimal bandwidth allocation before and after replacement, respectively. Therefore, it can be shown that

(33) $$|x_{new}^{*}\left(C\right)|\leq |x_{old}^{*}\left(C\right)|,\;\mathrm{when}\;y\geq T\;\mathrm{and}\;(x_{q},y_{q})\in M.$$

Consequently, when the new response is positive, and the selected old record $(x_{q},y_{q})\in M$, the proposed algorithm is self-aware after record replacement.

#### 4.2.2. New Negative Response

Define a set N as

(34) $$N=\{\left(x_{q},y_{q}\right):\left(x_{q},y_{q}\right)\in P,\;S_{4}\leq 0,\;\forall x\}.$$

We can then see that

(35) $$O_{new}\left(C\right)\subset O_{old}\left(C\right),\mathrm{when}\;y<T\;\mathrm{and}\;(x_{q},y_{q})\in N.$$

As a result,

(36) $$|x_{new}^{*}\left(C\right)|>|x_{old}^{*}\left(C\right)|,\mathrm{when}\;y<T\;\mathrm{and}\;(x_{q},y_{q})\in N.$$

Therefore, for the cases of new negative responses, when the old record to be replaced satisfies $(x_{q},y_{q})\in N$, the proposed algorithm is also self-aware.

#### 4.2.3. Hardware-Friendly Replacement Strategy

Although the self-awareness can be achieved by the proposed algorithm by record replacement, high computation complexities may be required for the search of old record $\left(x_{q},y_{q}\right)$ satisfying (33) or (36). This is because the search involves the computation of $S_{4}$ in (30) over all the x. To simplify the search operations, it can be shown from (30) that

(37) $$S_{4}>0,\;\forall x,\;\mathrm{when}\;y\geq T\;\mathrm{and}\;y_{q}<T,$$

(38) $$S_{4}\leq 0,\;\forall x,\;\mathrm{when}\;y<T\;\mathrm{and}\;y_{q}\geq T.$$

Therefore, when y≥T and $y_{q}<T$, then the record $\left\{x_{q},y_{q}\right\}$ belongs to M by (31) and (37). Likewise, the record $\left\{x_{q},y_{q}\right\}$ belongs to N for y<T and $y_{q}\geq T$ by (34) and (38). Based on the results, we can further derive from (33) and (36) that

(39) $$|x_{new}^{*}\left(C\right)|\leq |x_{old}^{*}\left(C\right)|,\;y\geq T\;\mathrm{and}\;y_{q}<T,$$

(40) $$|x_{new}^{*}\left(C\right)|>|x_{old}^{*}\left(C\right)|,\;y<T\;\mathrm{and}\;y_{q}\geq T.$$

Only simple comparisons are necessary for (39) and (40) for the selection of record $\left\{x_{q},y_{q}\right\}$ achieving QoS awareness without the computation of $S_{4}$ values.

Given a new record $\left\{x^{*},y\right\}$, there may exist more than one old record satisfying (39) or (40). In this study, we select the old record as the replacement target in the First-In First-Out (FIFO) fashion. Define

(41) $$Q=\left\{\begin{array}{cc} \{\left(x_{r},y_{r}\right):y_{r}<T,\left(x_{r},y_{r}\right)\in P\}, & \mathrm{when}\;\;\;y\geq T,\\ \{\left(x_{r},y_{r}\right):y_{r}\geq T,\left(x_{r},y_{r}\right)\in P\}, & \mathrm{when}\;\;\;y<T. \end{array}\right.$$

The target record to be replaced $\left(x_{q},y_{q}\right)$ is then the oldest record in Q. In this way, the most recent records will be kept in the profile for accurate QoS prediction. Algorithm 2 summarizes the corresponding record replacement and profile updating schemes for attaining QoS awareness.
**Algorithm 2** The Profile Updating Algorithm 1: **procedure**
Profile_Update($x^{*}$, y, P, *p*, *C*, *T*) 2:     **if** p<C **then** 3:         p←p+1. 4:     **else** 5:         Determine set Q by (41). 6:         **if** Q≠∅ **then** 7:             $(x_{q},y_{q})\leftarrow \mathrm{oldest}\mathrm{record}\mathrm{in}\;Q$. 8:         **else** 9:             $(x_{q},y_{q})\leftarrow \mathrm{oldest}\mathrm{record}\mathrm{in}\;P$. 10:         **end if** 11:         $P\leftarrow P\setminus \{x_{q},y_{q}\}$. 12:     **end if** 13:     $P\leftarrow P\cup \{x^{*},y\}$. 14:     **return** P, *p*. 15: **end procedure**


## 5. Proposed FPGA Accelerator for QoS Management

It is usually desired to employ a SoC for the QoS management in a LAN because of low power consumption and low deployment costs. However, because the processor of the SoC may have only limited computation capacities, the computation time of the software implementation of the algorithm in the SoC is high. By adopting the dedicated hardware circuits as the accelerator for the processor, we are then able to achieve realtime QoS management for the GRNN-based delivery quality prediction with dynamic profile updating.

**Figure 2:**
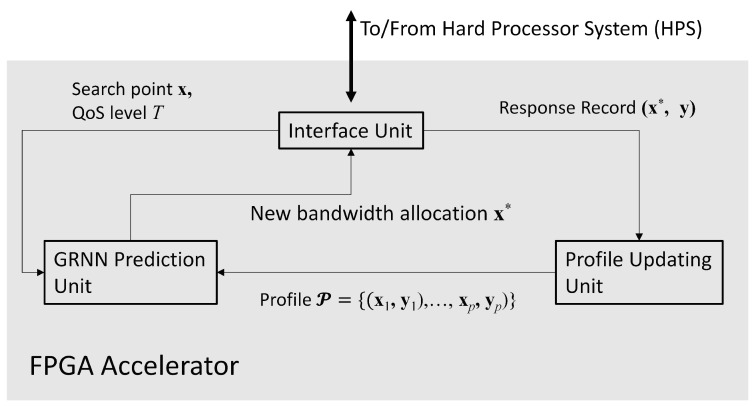
The architecture of the proposed FPGA accelerator. It can be separated into three parts: the GRNN prediction unit, the profile updating unit, and the interface unit.

As shown in Figure 2, the proposed FPGA accelerator contains three parts: the GRNN prediction unit, the profile updating unit, and the interface unit. The interface unit is designed for the interaction with the processor of the SoC. The interface unit has simple architecture mainly containing buffers. The processor of the SoC is able to access the buffers in the interface unit for providing source data and collecting computation results from the accelerator. The goal of the GRNN unit is carry out the computation of (13) by FPGA. The profile updating unit is responsible for performing the record replacement operations in Algorithm 2. In the following subsections, we focus on the discussions of GRNN prediction unit and the profile updating unit.

### 5.1. GRNN Prediction Unit

**Figure 3:**
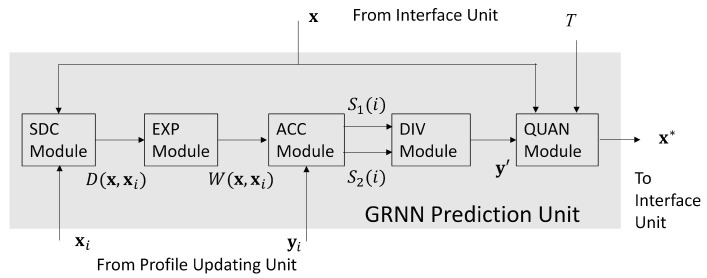
The architecture of the GRNN prediction unit.

The GRNN prediction unit is a hardware implementation of operations in (13). Based on the profile P provided by the profile updating unit and the QoS level *T* provided by the interface unit, the goal of GRNN unit is to search for $x^{*}$, the optimal bandwidth allocation for the service. As revealed in Figure 3, there are 5 modules in the GRNN unit, which are termed the SDC (e.g., Squared Distance Computation), the EXP (e.g., EXPonent), the ACC (e.g., ACCumulation), the DIV (e.g., DIVision), and the QUAN (e.g., QUANtization) modules, respectively.

**Figure 4:**
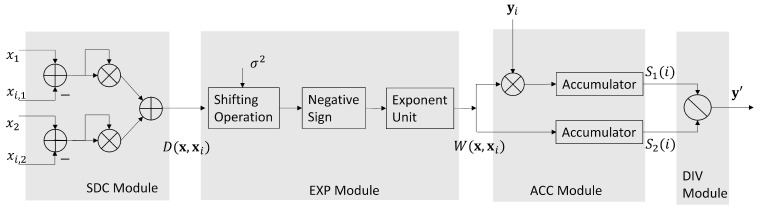
The architectures of the SDC module, EXP module, ACC module and DIV module.

Given a candidate x∈B, the goal of SDC module and EXP module are to compute $D(x,x_{i})$ in (10) and $W(x,x_{i})$ in (9), respectively. Specifically, the computations of ${\left(x_{j}-x_{i,j}\right)}^{2}$ in (10) are carried out in the SDC module. In this study, the core network contains only 2 links (i.e., n=2). Therefore, we need only two Floating Point (FP) multipliers and three FP adders in the SDC module. In the FP arithmetic operators, all the numbers are in IEEE 754 single precision format [23]. In the EXP module shown in Figure 4, there is only a single FP exponent computation unit for the computation of $W(x,x_{i})$. The $\sigma^{2}$ in (9) is chosen as a power of 2 so that the division operation for $W(x,x_{i})$ is equivalent to simple shifting operations.

The ACC module is responsible for the computations of both $\sum_{i=1}^{p}y_{i}W\left(x,x_{i}\right)$ and $\sum_{i=1}^{p}W\left(x,x_{i}\right)$. Note that the EXP module only provides $W(x,x_{i})$ for i=1,…,p, sequentially. As a result, the ACC module offers the accumulation of the partial sums $S_{1}\left(i\right)$ and $S_{2}\left(i\right)$, defined as

(42) $$S_{1}\left(i\right)=\sum_{k=1}^{i}y_{i}W\left(x,x_{k}\right),\;\;S_{2}\left(i\right)=\sum_{k=1}^{i}W\left(x,x_{k}\right).$$

In the ACC module, the computation of $S_{1}\left(i\right)$ and $S_{2}\left(i\right)$ are performed by separate FP accumulators. When i=p, the $S_{1}\left(p\right)$ and $S_{2}\left(p\right)$ can serve as the inputs to the DIV module for the computation of $y^{\prime }$.

**Figure 5:**
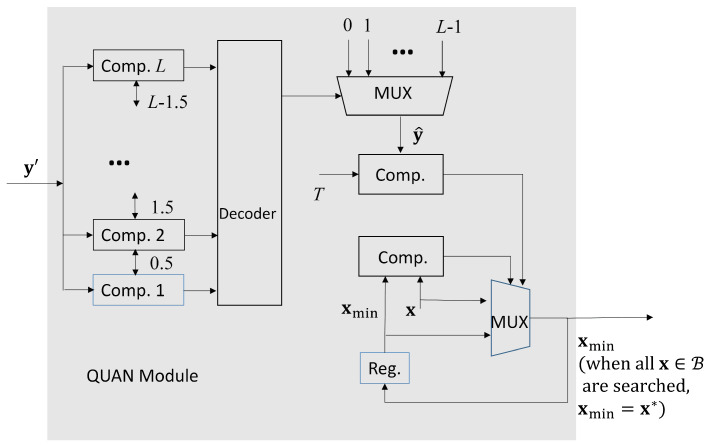
The architecture of the QUAN module.

There is only a single FP divider for the computation of $y^{\prime }$ in (8) in the DIV module. From Figure 5, we see that the $y^{\prime }$ is further processed by QUAN module. It then produces the final result $\hat{y}$ in (11). As shown in Figure 5, we let $x_{\min}$ be the current $x^{*}$. When $\hat{y}>T$, and $\left|x|<\right|x_{\min}|$, then $x_{\min}$ is updated as x. When all the x in B is searched, the final $x_{\min}$ is the final bandwidth allocation result $x^{*}$.

**Figure 6:**
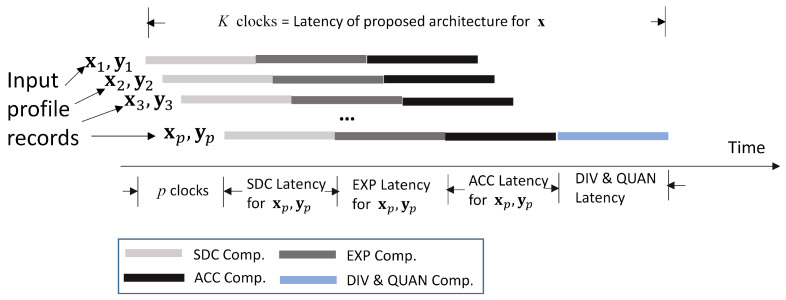
The pipeline operations for the GRNN prediction unit.

Given a bandwidth allocation x, an advantage of the proposed architecture is that the SDC, EXP, and ACC modules are operating in a pipelined fashion for enhancing the throughput for GRNN computation. As shown in Figure 6, given x, profile records $\left(x_{i},y_{i}\right)$, i=1,…,p, are fetched one at a time. The adders and multipliers in the SDC module are pipelined. Therefore, for different profile records, $D(x,x_{i})$ can be computed concurrently. Likewise, the exponent computation unit in the EXP module is pipelined. The $W(x,x_{i})$ for different profile records are also computed in an overlapping fashion. The multiplication and accumulation operations can also be carried out in parallel in the ACC module. Let *K* be the latency for updating $x_{\min}$ from x. It can then be observed from Figure 6 that *K* is given by

(43) $$K=p+K_{\mathrm{SDC}}+K_{\mathrm{EXP}}+K_{\mathrm{ACC}}+K_{\mathrm{DIV}}+K_{\mathrm{QUAN}}$$

the latency $K_{\mathrm{SDC}}$, $K_{\mathrm{EXP}}$, $K_{\mathrm{ACC}}$, $K_{\mathrm{DIV}}$ and $K_{\mathrm{QUAN}}$ are independent of the profile size *p*. They are determined by the latency of FP adders, multipliers, comparators, exponent operators, and/or dividers. Therefore, the latency *K* only grows linearly with *p*.

The pipelined operations can also be extended for different search candidates $x^{\prime }s$∈B. Let *J* be the number of search candidates. The number of candidates is dependent on the search step size Δ and the search algorithm [13]. Furthermore, $t_{1}$ be the total latency for finding $x^{*}$. Let $\bar{t}$ be average latency per search candidate. That is,

(44) $$\bar{t}=\frac{t_{1}}{J}.$$

When operations for different search candidates are not overlapping, $t_{1}=J\times K$. In this case, (44) decreases to $\bar{t}=K$.

### 5.2. Profile Updating Unit

The profile updating unit contains the profile $P=\{\left(x_{i},y_{i}\right),i=1,\;\ldots ,\;p\}$. Furthermore, the unit is responsible for updating P based on Algorithm 2. Recall that the set Q⊆P defined in (41) plays an important role for the record replacement by Algorithm 2. In the profile updating unit, Q can be easily identified. The oldest record in Q can also be easily removed. These advantages facilitate the updating process for the profile.

**Figure 7:**
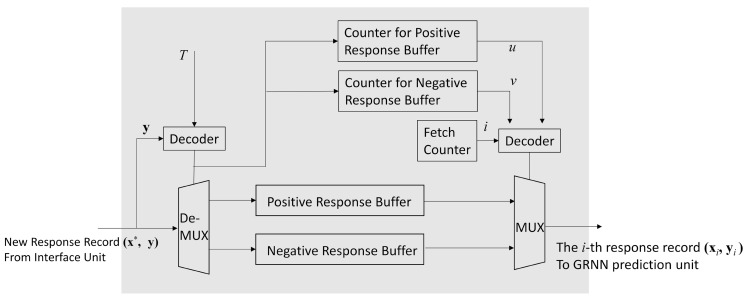
The architecture of the profile updating unit. In the architecture, both the positive response buffer and negative response buffer are used for storing response records in P.

As shown in Figure 7, there are two buffers in the profile updating unit: the positive response buffer, and the negative response buffer. Each record $(x_{i},y_{i}),i=1,\;\ldots ,\;p,$ in the profile P is assigned to one of the buffers. Given a threshold T>0, a record $\left(x_{i},y_{i}\right)$ is assigned to the positive response buffer when $y_{i}\geq T$. Otherwise, the record is assigned to the negative response buffer.

**Figure 8:**
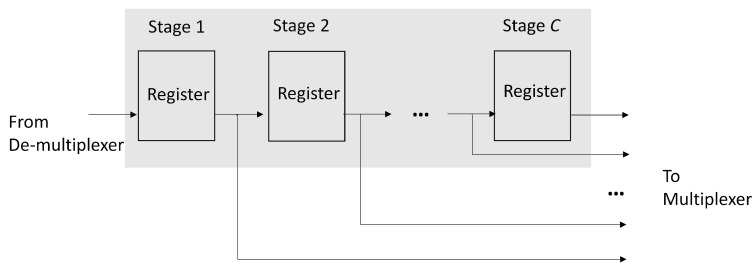
The architecture of positive response buffer and negative response buffer. Each buffer is a *C*-stage shift register supporting SIPO operations.

Both the positive response buffer and negative response buffer have the same architecture, as revealed in Figure 8. We can see from Figure 8 that the each buffer is a *C*-stage shift register supporting serial-in parallel-out (SIPO) operations, where *C* is the upperbound of the profile size. Therefore, each buffer accepts at most one response record at a time. All the registers in the buffers are connected to the output multiplexer shown in Figure 7. In this way, the content of each register of the buffers can be easily fetched.

Because the actual profile size p<C, some stages in the shift register may be empty, or contain invalid profile record. To facilitate the profile updating process, each buffer in the profile updating unit is associated with a counter. The value of each counter indicates the number of valid records in the corresponding buffer. Let *u* and *v* be the value of the counter associated with positive response buffer and negative response buffer, respectively. Therefore, u+v=p, u≥0, and v≥0. In addition, the *u* valid records and *v* valid records are located in the first *u* stages and the first *v* stages of the shift registers in positive response buffer and negative buffer, respectively. Only the stages with valid records in the shift registers are accessed by the GRNN prediction unit.

Given a newly received record $\left(x^{*},y\right)$ for updating the profile P, two cases are considered separately in the profile updating unit: p<C and p=C.

#### 5.2.1. Updating Buffers in the Profile Updating Unit for p<C

In this case, only record appending is necessary. Dependent on the value of y, the newly received record $\left(x^{*},y\right)$ is appended to the positive response buffer or negative response buffer. When y≥T, the record $\left(x^{*},y\right)$ is assigned to the positive response buffer. In addition, both *p* and *u* are incremented by 1, and *v* remains the same. When y<T, we append $\left(x^{*},y\right)$ to the negative response buffer. Both *p* and *v* are incremented by 1, and *u* remains the same.

**Figure 9:**
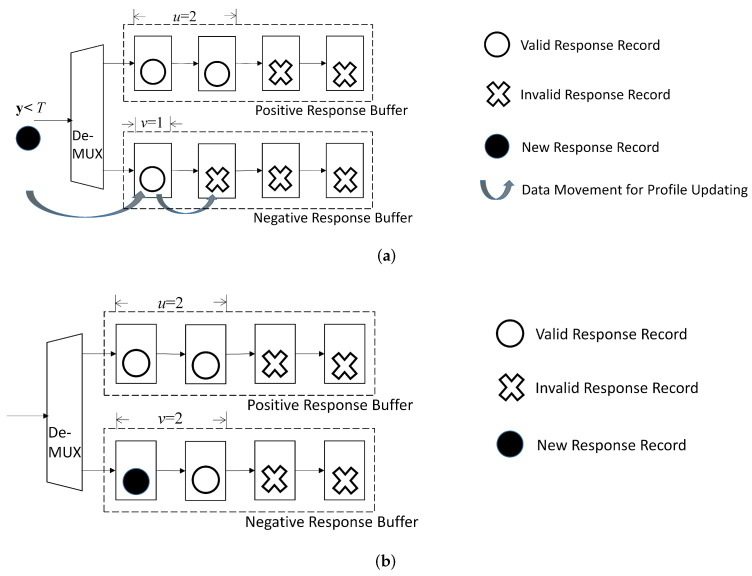
A simple example for updating buffers in profile updating unit for p<C. In this example, C=4, p=3, u=2, and v=1 before updating. Assume y<T for the new record. The new record is then assigned to negative response buffer. After updating, p=4, u=2, and v=2. (**a**) Before updating; (**b**) After updating.

Figure 9 shows a simple example for the corresponding operations, where C=4, p=3, u=2, and v=1 before the updating. It is assumed y<T in this example. As a result, $\left(x^{*},y\right)$ is assigned to the negative response buffer. That is, after the updating, p=4, u=2, and v=2.

#### 5.2.2. Updating Buffers in the Profile Updating Unit for p=C

The record replacement is required in this case because the size of the profile has already attained its upperbound. To carry out the replacement operations, the set Q∈P should be first found, as shown in Algorithm 2. The oldest record in Q is subsequently removed. The newly received record $\left(x^{*},y\right)$ is then appended to the profile P.

From (41), we observe that the set Q can be easily identified based on positive response buffer and negative response buffer. When y≥T, the set Q is the negative response buffer by (41). The *v*-th stage in the shift register of negative response buffer contains the oldest record. It is then removed. The record $\left(x^{*},y\right)$ is assigned to the positive response record. After the replacement operations, the profile size *p* remains the same. However, the value of *v* is decreased by 1 because of the removal operation for the negative response buffer. Furthermore, since the new record is appended to the positive response record, we increase the *u* by 1.

By contrast, when y<T, the set Q is the positive response buffer. The record located at the *u*-th stage of the shift register of the positive response buffer is the oldest record, and is removed. The record $\left(x^{*},y\right)$ is appended to the negative response record. Therefore, *u* and *v* are incremented by 1 and decremented by 1, respectively. The profile size *p* remains the same.

**Figure 10:**
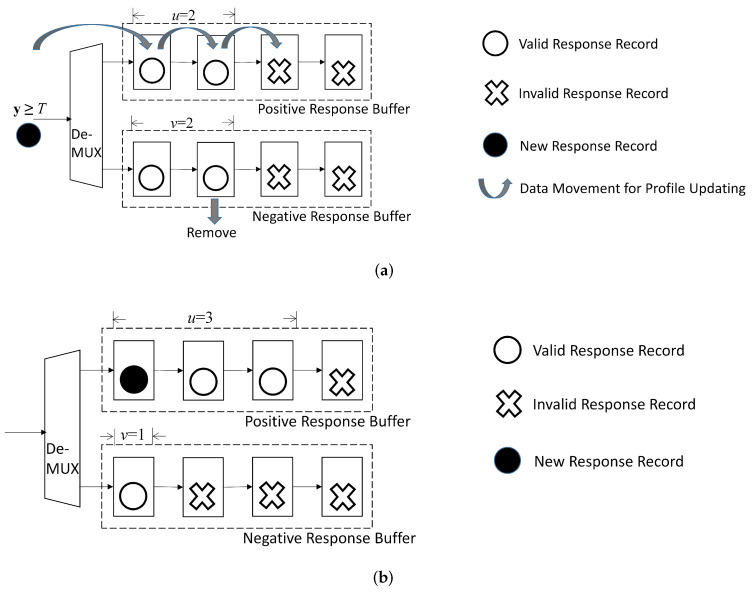
A simple example for updating buffers in profile updating unit for p=C. In this example, p=4, C=4, u=2 and v=2 before updating. Assume y≥T for the new record. The new record is then assigned to positive response buffer. After updating, p=4, u=3, and v=1. (**a**) Before updating; (**b**) After updating.

An example of record replacement for p=C=4 is provided in Figure 10. In this example, u=v=2 before updating. Furthermore, the new record $\left(x^{*},y\right)$ with y≥T is considered. The set Q is then the negative response buffer. Because v=2 before updating, the record in the second cell of the negative response buffer is removed. The new record $\left(x^{*},y\right)$ is assigned to the positive buffer. Consequently, after the record replacement, u=3, v=1. Furthermore, because the profile size remains the same, p=4 after record replacement.

## 6. Experimental Results

This section presents some experimental results of the proposed smart SoC, which have been deployed in the real LAN for QoS management. The setup for the experiments is first provided in detail. This is followed by the evaluations of hardware costs and computation speed of the SoC. The performance metrics of the SoC for QoS management in terms of DLR and RAB for different services are subsequently included with comparisons.

### 6.1. Experimental Setup

**Figure 11:**
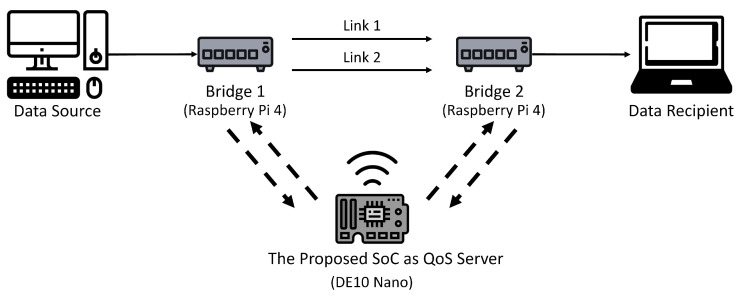
The real LAN for the experiments. The proposed SoC is deployed as the QoS server for the LAN.

As shown in Figure 11, the LAN for the experiments contains 2 bridges, 1 QoS server, and a single core network. There are two links (i.e., n=2) in the core network. Each link is a Gigabyte Ethernet. The communication between the QoS server and each bridge is by WiFi. The ERAB measurements for a service are carried out by the Bridge 1 or Bridge 2 depending on the location of the source. The corresponding ERAB reports are then sent to the QoS server. Upon receiving the reports, the QoS server computes the new bandwidth allocation for the service. The new allocation is subsequently sent to the corresponding bridge for traffic control operations.

Raspberry Pi 4 computers are adopted for the implementation of Bridge 1 and Bridge 2. The QoS server is built on Terasic DE-10 Nano board. The FPGA device for the DE-10 Nano board is Intel Cyclone V 5CSEBA6. The HPS associated with the DE-10 board is based on ARM Cortex A9 processor with 800 MHz clock rate The proposed FPGA accelerator has been simulated, implemented and mapped in FPGA using Cyclone V 5CSEBA6. The ModelSim is the simulator for the RTL level verification. Furthermore, the Qsys is used for building the SoC for the evaluation of the proposed algorithms and architectures for QoS management. The FPGA accelerator operates at maximum clock rate of 50 MHz.

The virtual switch in each bridge is implemented by the Open vSwitch (OVS) [24]. In our experiments, the virtual switch is adopted for link aggregation and traffic shaping. Let $r_{j}$ be the source data rate assigned to the *j*-th link of the core network. In the proposed link aggregation scheme, $r_{j}$ is computed by

(45) $$r_{j}=R\frac{x_{j}}{\left|x\right|},$$

where *R* is the total source data rate. The flow tables for packet matching operations in the virtual switch is used for traffic shaping. The matching rules for the flow tables are updated by the SDN controller in the QoS server. An Openflow controller operating in accordance with the bandwidth allocation results from the proposed algorithm is adopted as the SDN controller. The OpenFlow protocol [3] is used for the delivery of commands produced by the SDN controller. In our experiments, there are twelve response levels (i.e., L=12) for the QoS management. We set $\left\{\eta_{1},\;\ldots ,\;\eta_{11}\right\}=\left\{-11.25,-8.75,-6.25,-3.75,-1.25,1.25,3.75,6.25,8.75,11.25,13.75\right\}$ (in Mbps) for converting the ERAB to service quality y by (6). For the search space B for each service, we set the step size Δ=0.25 Mbps in (7). Furthermore, both Link 1 and link 2 have the same maximum bandwidth $B_{1}=B_{2}=40$ Mbps.

### 6.2. Hardware Costs and Computation Speed

In the proposed FPGA accelerator, both the arithmetic operators and memory buffers are the major contributing factors to the utilization of hardware resources. The arithmetic operators are the FP adders, FP multipliers, FP accumulators, exponent operators, FP dividers, and FP comparators. The memory buffers are the shift registers. Table 3 shows the asymptotic analysis of number of arithmetic operators, and the size of shift registers. The analysis is against the profile size upper bound *C*, the number of links *n*, and the number of service quality levels *L* for the GRNN prediction unit and profile updating unit. The analysis is based on the big-*O* function.

It can be observed from Table 3 that the number of FP accumulators, exponent operators, FP dividers are independent of *C*, *n* and *L*. This is because only 2 FP accumulators, 1 exponent operator, and 1 FP divider are used for GRNN prediction, as revealed in Figure 4. Because all the FP operators are pipelined, we can see from Figure 6 the latency for GRNN prediction may still be low even for large profile size.

We see from Table 3 that the number of FP adders and FP multipliers grows with the number of links *n* because of the squared distance computation in SDC unit. We also conclude from Table 3 that the number of FP comparators increases linearly with *L* for the quantization operations in QUAN unit shown in Figure 5. Furthermore, it can be observed from Table 3 that the size of shift registers grows with *C* and *n*. This is because the shift registers are used for the implementation of positive response buffer and negative response buffer, as shown in Figure 8. It may not be necessary to specify large number of links *n* and/or high number of quality levels *L*. However, it is usually desired to have a high upper bound of profile size *C* so that robust GRNN prediction could be achieved.

Table 4 shows the utilization of FPGA resources of the proposed architecture for various upper bound *C* to profile sizes. The area costs considered in the table are Adaptive Logic Modules (ALMs), dedicated registers, embedded memory bits and DSP blocks. It can be observed from Table 4 that the number of DSP blocks is independent of *C*. This is because the DSP blocks are mainly used for the implementation of arithmetic operators. Both ALMs and dedicated registers are used for the implementation of buffers for the profile updating unit. Therefore, their utilizations grow with *C*. In fact, when C=360, the proposed architecture consumes 36,462 ALMs and 84,008 dedicated registers, respectively. The target FPGA device Cyclone V 5CSEBA6 on Terasic DE-10 Nano FPGA board contains 41,910 ALMs, 167,640 registers, 5,662,720 block memory bits, and 112 DSP blocks. Therefore, when C=360, the proposed circuit consumes 87.00% of ALMs, 50.11% of registers, 0.15% of block memory bits, and 18.75% of DSP blocks of the target FPGA device. That is, the proposed SoC with large profile size can still be accommodated in the light-weight FPGA devices for QoS management.

In addition to the area costs, the computation speed is an important concern for SoC implementation. There are three speed measurements considered in this study. Recall from (44) that $\bar{t}$ is the average latency per search candidate, given a profile P. Furthermore, $t_{1}$ is the total latency for finding the optimal bandwidth allocation $x^{*}$ over search space B. The first and the second speed measurements are $\bar{t}$ and $t_{1}$, respectively. For our experiments, the number of candidates *J* in (44) is found by the subspace search algorithm in [13]. The third speed measurement is the latency for updating profile P given a new response record $\left(x^{*},y\right)$, denoted by $t_{2}$. The latency for profile updating $t_{2}$ is not a part of the latency $t_{1}$. The measurements of $t_{1}$ and $t_{2}$ are carried out independently.

Table 5 reveals the latency $\bar{t}$ of the proposed SoC. To evaluate the proposed architecture, the latency of some existing GRNN hardware architectures is also included in Table 5. Even with higher profile size, we can see from Table 5 that the proposed architecture has comparable latency to the architecture in [13], which is also based on pipelined operations. Furthermore, as compared with the architecture in [21], the proposed architecture has lower latency. Although architectures in [20,22] have faster computation speeds, their profile sizes are small, and may not be suitable for accurate delivery quality prediction. The proposed architecture has efficient computation performance because it is based on pipelined operations. The parallel operations for different search candidates and response records are beneficial for enhancing the computation efficiency even with large profile size.

Table 6 and Table 7 show the latencies $t_{1}$ and $t_{2}$ of the proposed SoC for various profile sizes *p*, respectively. For comparison purpose, the $t_{1}$ and $t_{2}$ measured from software-based systems running on a personal computer (PC) with Intel I5 CPU operating at 2.90 GHz are also reported. It can be observed from Table 6 and Table 7 that the latencies of the proposed SoC for bandwidth allocation and profile updating are significantly lower than their software counterparts. Although the latency $t_{1}$ increases with profile size *p* for both SoC and software-based implementations, only slow growth is observed for the SoC because of the pipelined operations for the GRNN computation. By contrast, surge in computation time occurs for the software-based system. As a result, the speedup of the proposed SoC over its sofware counterpart for $t_{1}$ computation increases with profile size *p*.

Because of the simplicity of Algorithm 2, we can observe from Table 7 that both the proposed SoC and its software counterpart have stable latency $t_{2}$ for profile updating as the profile size *p* increases. It can also be seen from Table 7 that the latency $t_{2}$ is only 0.12 ms for the proposed SoC. The speeup of the proposed SoC is still above 10 over its software counterpart.

### 6.3. Bandwidth Allocation, DLR and RAB

**Figure 12:**
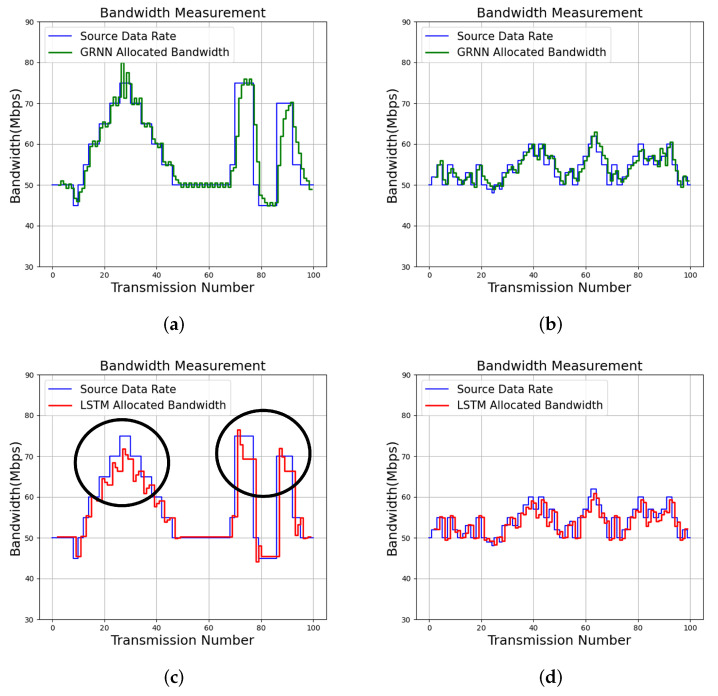
Bandwidth allocation results of the proposed algorithm with T=6 and LSTM [16] for various services. Because LSTM may not be self-aware, parts of bandwidth allocation results where self-awareness are not attained are marked. (**a**) Proposed GRNN for Service 1; (**b**) Proposed GRNN for Service 2; (**c**) LSTM for Service 1; (**d**) LSTM for Service 2.

The input source data rates can be tracked by the proposed algorithm for effective bandwidth allocation, as shown in Figure 12. The profile size constraint for the experiments is C=80. We can see from Figure 12 that QoS management results for two services (termed Service 1 and Service 2) with QoS level T=6 are evaluated. Each service can be divided into 100 transmissions, where each transmission is associated with different time slots. The proposed algorithm is adopted for the QoS management for each transmission. The source data packets in the experiments are produced by iPerf [25].

For comparison purposes, the tracking results of the input source data rates by long short term memory (LSTM) [16] are also included in Figure 12. The LSTM algorithm [14,16] is a neural network capable of exploring the temporal correlation of input source data for prediction. The offline training operations for the LSTM algorithm are carried out by NVIDIA Geforce GTX 1060 GPU. In contrast, no offline training operations are required by the proposed algorithm. In addition, the average DLR and RAB over 100 transmissions for each service for various algorithms are included in Table 8. The measurements of RAB and DLR for each transmission are by (2) and (3), respectively.

We can observe from Figure 12 that the proposed algorithm is effective for tracking the source data rates. To elaborate this fact, as shown in Figure 12, the proposed algorithm will allocate more bandwidths to a service when the deficiency of bandwidth to the service has been observed. In addition, it may reduce the bandwidth when the excessive bandwidth is assigned to to service. These results are consistent with the analytical results shown in (22), (25), (39), and (40). By contrast, the LSTM algorithm may not be self-aware. Examples revealing non-awareness for QoS management are exposed in the marked results in Figure 12, where bandwidth to a service is removed by LSTM even in case of deficiency.

**Figure 13:**
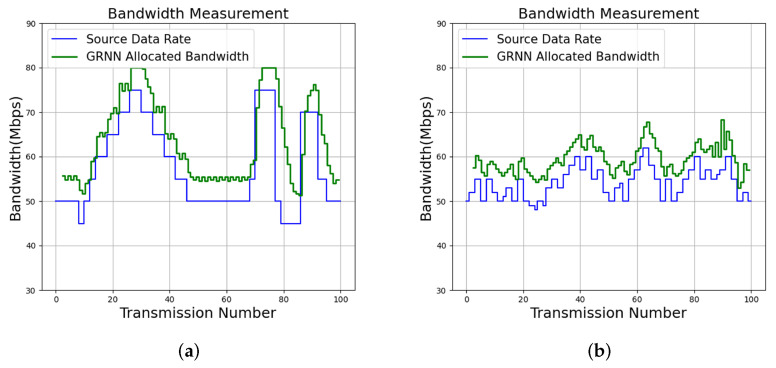
Bandwidth allocation results of the proposed algorithm with T=8 for various services. (**a**) Proposed GRNN for Service 1; (**b**) Proposed GRNN for Service 2.

Because the proposed algorithm is self-aware, it has low RAB and DLR for tracking source data rates, as revealed in Table 8. Furthermore, when the DLR is an important concern, the proposed algorithm is able to further lower the DLR by increasing the QoS level *T*. In addition to QoS level T=6, Table 8 and Figure 13 show the results of the proposed algorithm with QoS level T=8 for Service 1 and Service 2. We can observe from Table 8 and Figure 13 that the DLR values for each service are effectively reduced by allocating more bandwidth for that service. In fact, when T=8, the DLR for Service 1 and Service 2 are 0.02 Mbps and 0.00 Mbps, respectively. These results confirm that higher QoS levels are beneficial for data delivery when low DLR values are desired.

To evaluate the impact of the performance of the proposed algorithm on the upper bound of profile size *C*, Table 9 shows the average RAB and DLR for different upper bound on profile sizes C=30, C=50 and C=80, respectively. Both Service 1 and Service 2 are considered in the experiment. For each service, the performance of two QoS levels T=6 and T=8 are reported. We observe from Table 9 that the proposed QoS management algorithm based on larger upper bound *C* has lower average RAB and average DLR values. This is because larger number of past response records are available for more accurate quality prediction. Furthermore, given *C*, lower average DLR values can be attained by adopting QoS level with higher *T* at the expense of larger average RAB values. While attaining accurate tracking for input source data rates, the proposed algorithm is able to provide high flexibilities for QoS management by specifying different upper bound for profile sizes and QoS levels for data delivery.

## 7. Conclusions

A smart SoC has been successfully deployed for QoS management in a virtual LAN. An FPGA accelerator has been implemented for the GRNN-based service quality prediction so that the bandwidth allocated for a service can be optimized with low computation latency. Both analytical and numerical studies have been provided for demonstrating the self-awareness of the proposed algorithm for QoS management. Subject to the constraint on the profile size, the analytical study shows that the proposed profile updating algorithm is still able to maintain self-awareness. Numerical results reveal that the proposed FPGA accelerator utilizes only limited hardware resources, even for large profile size upper bounds. When applied for QoS management, the SoC based on the FPGA as an accelerator has low latency for finding the optimal bandwidth allocation and profile updating. The proposed SoC therefore is beneficial as a hardware VNF for effective QoS management over virtual LAN with low implementation costs.

## Figures and Tables

**Table 1:** An example of service qualities and their corresponding ERAB intervals. In this example, the network system has six service quality levels (i.e., L=6). The set of thresholds is given as $\left\{\eta_{1},\;\ldots ,\;\eta_{5}\right\}=\{$1.25, 3.75, 6.25, 8.75, 11.25}.

Service Quality y	ERAB Intervals	Interval Range (Mbps)
5	$I_{5}$	[11.25,∞)
4	$I_{4}$	[8.75,11.25)
3	$I_{3}$	[6.25,8.75)
2	$I_{2}$	[3.75,6.25)
1	$I_{1}$	[1.25,3.75)
0	$I_{0}$	[−∞,1.25)

**Table 2:** An example of a set of QoS levels and their corresponding service qualities and ERAB intervals. This example is based on the service qualities and ERAB intervals defined in Table 1.

QoS Level with *T*	Allowed Service Qualities	Allowed ERAB Intervals
5	5	$I_{5}$
4	4, 5	$I_{4},\;I_{5}$
3	3, 4, 5	$I_{3},\;I_{4},\;I_{5}$
2	2, 3, 4, 5	$I_{2},\;I_{3},\;I_{4},\;I_{5}$
1	1, 2, 3, 4, 5	$I_{1},\;I_{2},\;I_{3},\;I_{4},\;I_{5}$

**Table 3:** Asymptotic analysis of number of arithmetic operators and the size of shift registers for the proposed FPGA architecture.

Unit Name	FP Adder	FP Mult.	FP Acc.	FP Divider	Exponent Operator	FP Comparator	Shift Register
GRNN Prediction Unit	O(n)	O(n)	O(1)	O(1)	O(1)	O(L)	0
Profile Updating Unit	0	0	0	0	0	0	O(nC)
Overall	O(n)	O(n)	O(1)	O(1)	O(1)	O(L)	O(nC)

**Table 4:** The utilization of FPGA resources of the proposed architecture for various upper bounds *C* of profile sizes.

Profile Size Upper Bound	30	50	80	150	300	360
Number of ALMs	9444	11,366	14,385	20,955	33,109	36,462
Number of Registers	19,105	23,521	30,372	46,185	75,091	84,008
Block Memory Bits	8768	8768	8768	8768	8768	8768
Number of DSP Blocks	21	21	21	21	21	21

**Table 5:** The average latency $\bar{t}$ for a single prediction by various GRNN hardware architectures.

Hardware Architecture	FPGA Device	Clock Rate	Profile Size	Average Latency $\bar{t}$
Arch. in [13]	Cyclone V 5CSEBA6	50 MHz	54	1.22 μs
Arch. in [20]	Virtex X2V1000	50 MHz	10	1.00 μs
Arch. in [21]	Spartan 3 XC3S2000	10 MHz	55	5.60 μs
Arch. in [22]	Cyclone III EP3C120	NA	16	0.74 μs
Proposed	Cyclone V 5CSEBA6	50 MHz	80	1.63 μs

**Table 6:** The total latency $t_{1}$ for finding the optimal bandwidth allocation $x^{*}$ for various profile sizes *p* over search space B given a profile P.

Profile Size	30	50	80	100	200	300	360
Proposed SoC	22.09 (ms)	28.31 (ms)	34.20 (ms)	38.56 (ms)	52.88 (ms)	64.51 (ms)	67.89 (ms)
Personal Computer	1169.68 (ms)	1864.86 (ms)	3418.17 (ms)	3500.69 (ms)	5700.90 (ms)	7487.50 (ms)	8030.31 (ms)
Speedup	52.95	65.87	99.95	90.79	107.81	116.07	118.28

**Table 7:** The latency $t_{2}$ for updating profile P with various profiles sizes *p* given a new response record $\left(x^{*},y\right)$.

Profile Size	30	50	80	100	200	300	360
Proposed SoC	0.11 (ms)	0.12 (ms)	0.12 (ms)	0.12 (ms)	0.12 (ms)	0.12 (ms)	0.12 (ms)
Personal Computer	1.73 (ms)	1.69 (ms)	1.59 (ms)	1.54 (ms)	1.61 (ms)	1.66 (ms)	1.64 (ms)
Speedup	15.72	14.08	13.25	12.83	13.41	13.83	13.67

**Table 8:** The average DLR and RAB values of the proposed GRNN algorithm with T=6 and T=8, and its LSTM [16] counterparts for source data rate prediction for various services.

Algorithms	LSTM [16]		Proposed (*T* = 6)		Proposed (*T* = 8)	
	ave RAB	ave DLR	ave RAB	ave DLR	ave RAB	ave DLR
Service 1 (Mbps)	0.82	2.28	1.08	0.88	5.35	0.02
Service 2 (Mbps)	1.38	1.62	1.12	0.90	5.32	0.00

**Table 9:** The average DLR and RAB values of the proposed GRNN algorithm with different profile size upper bounds *C* and different QoS levels *T*.

Profile Size Upper Bound		C=30		C=50		C=80	
		ave RAB	ave DLR	ave RAB	ave DLR	ave RAB	ave DLR
Service 1	T=6	1.84	1.19	1.02	1.06	1.08	0.88
(Mbps)	T=8	5.73	0.09	5.63	0.02	5.35	0.02
Service 2	T=6	1.30	0.68	1.28	0.88	1.12	0.90
(Mbps)	T=8	5.58	0.01	5.61	0.01	5.32	0.00

## Data Availability

The data are contained within the article.

## Abbreviations

The following abbreviations are used in this manuscript:

ACC	ACCumulation
ALM	Adaptive Logic Module
ARIMA	Auto-Regressive Integrated Moving Average
DIV	Division
DLR	Data Loss Rate
ERAB	Extended Residual Allocation Bandwidth
EXP	Exponent
FP	Floating Point
FPGA	Field Programmable Gate Array
GRNN	General Regression Neural Network
HPS	Hard Processor System
LAN	Local Area Network
LSTM	Long Short Term Memory
MLP	Multilayer Perceptron
NIC	Network Interface Card
NFV	Network Function Virtualization
PC	Personal Computer
OVS	Open vSwitch
QUAN	Quantization
QoS	Quality of Service
RAB	Residual Allocation Bandwidth
RNN	Recurrent Neural Network
SDN	Software-defined Networking
SIPO	Serial-In Parallel-Out
SoC	System on Chip
SDC	Squared Distance Computation
UDP	User Datagram Protocol
VNF	Virtual Network Function

## Appendix A. Frequently Used Symbols

**Table A1:** A list of frequently used symbols in this study.

Δ	Step size for the search of optimal bandwidth allocation.
$\eta_{k}$	$\left\{\eta_{1},\ldots ,\eta_{L-1}\right\}$ is the set of threshold for determining ERAB intervals $I_{k},$ k=0,…,L−1.
B	Set of all possible bandwidth allocations for the service.
$B_{j}$	Maximum allowed bandwidth at the link *j* for the service.
*C*	Upper bound of profile size *p*.
$I_{k}$	ERAB interval for service quality y=k.
*J*	Number of search candidates for GRNN prediction.
*L*	Number of service quality levels.
*n*	Number of links in the core network.
O	Set of bandwidth allocations x whose service quality prediction $\hat{y}$ is larger or equal to *T*.
P	Profile for GRNN prediction.
*p*	Number of records in the profile P.
Q	Set of records in P that can be replaced without losing QoS self-awareness.
*R*	Source data rate.
*T*	Lower bound of the service quality.
$\bar{t}$	The average latency per search candidate x. $\bar{t}=\frac{t_{1}}{J}$.
$t_{1}$	The latency for finding the optimal bandwidth allocation $x^{*}$ given profile P.
$t_{2}$	The latency for updating profile P given new response record $\left(x^{*},y\right)$.
*u*	The number of valid response records in the positive response buffer.
*v*	The number of valid response records in the negative response buffer.
x	A bandwidth allocation for the service.
|x|	Total bandwidth of the bandwidth allocation x.
$x^{*}$	Result of optimal bandwidth allocation.
$x_{i}$	$\left(x_{i},y_{i}\right)$ is the *i*-th record in profile P, where $x_{i}$ is the bandwidth allocation of the record.
$x_{j}$	The bandwidth of the *j*-th link.
y	Measured service quality for a bandwidth allocation.
$y^{\prime }$	Result of GRNN computation.
$\hat{y}$	Predicted service quality based on a bandwidth allocation x.
$y_{i}$	$\left(x_{i},y_{i}\right)$ is the *i*-th record in profile P, where $y_{i}$ is the measured service quality for $x_{i}$.
